# Q Fever Endocarditis and New *Coxiella burnetii* Genotype, Saudi Arabia

**DOI:** 10.3201/eid2004.131603

**Published:** 2014-04

**Authors:** Emmanouil Angelakis, Sameer Johani, Azeem Ahsan, Ziad Memish, Didier Raoult

**Affiliations:** Unité de Recherche.sur les Maladies Infectieuses et Tropicales Émergentes, Marseille, France (E. Angelakis, D. Raoult);; King Saud Bin Abdul Aziz University for Health Science, Jeddah, Saudi Arabia (S. Johani, D. Raoult);; King Fahd Medical City, Riyadh, Saudi Arabia (A. Ahsan);; Ministry of Health, Riyadh (Z. Memish)

**Keywords:** *Coxiella burnetii*, Q fever, Saudi Arabia, multispacer sequence typing, genotype, bacteria, zoonoses

**To the Editor.** Q fever is a worldwide zoonosis caused by an obligate intracellular bacterium, *Coxiella burnetii* (*1*). Q fever endocarditis is associated with surgery for 15%–73% of patients, causes death for 5%–65% of patients, and induces a large number of relapses when the endocarditis is inadequately treated (*1*). The most serious risk factor for endocarditis is a substantial underlying valvulopathy, but progression to endocarditis is also found in patients with clinically silent, previously undiagnosed, valvulopathies (*1*). Since the 1960s, Q fever has been recognized as a public health problem in Saudi Arabia, and studies have shown that coxiellosis occurs in livestock (*2*,*3*). Only a few cases of Q fever endocarditis in Saudi Arabia have been reported (*4*–*6*). We report 2 new cases of Q fever endocarditis and detection of a new *C. burnetii* genotype in this country.

The first case was detected in 2007 in a 45-year-old man in Saudi Arabia who had fever, pneumonia, and asthenia. A transesophageal echocardiogram showed endocarditis. Results of an immunofluorescence assay were positive for *C. burnetii*; phase I titers for IgG, IgM, and IgA were 51,200, 100, and 25, respectively, and phase II titers were 102,400, 200, and 50, respectively. Serum and blood samples were negative for *C. burnetii* by real-time PCR for the IS1111 and the IS30A spacers (*7*). For each sample, the quality of DNA extraction was verified by real-time PCR for a housekeeping gene encoding β-actin (*7*). The aortic valve was surgically replaced, and *C. burnetii*–specific PCR results for the valve were positive. According to multispacer sequence typing (*8*), this *C. burnetii* isolate was a new genotype, MST51 (Figure). A *C. burnetii* isolate was cultured from the valve of this patient by the shell-vial method that used human embryonic lung cells (*7*). IgG anticardiolipin testing results were negative (*9*). The patient was given 200 mg oral doxycycline daily and 200 mg oral hydroxychloroquine 3 times daily for 18 months.

**Figure:**
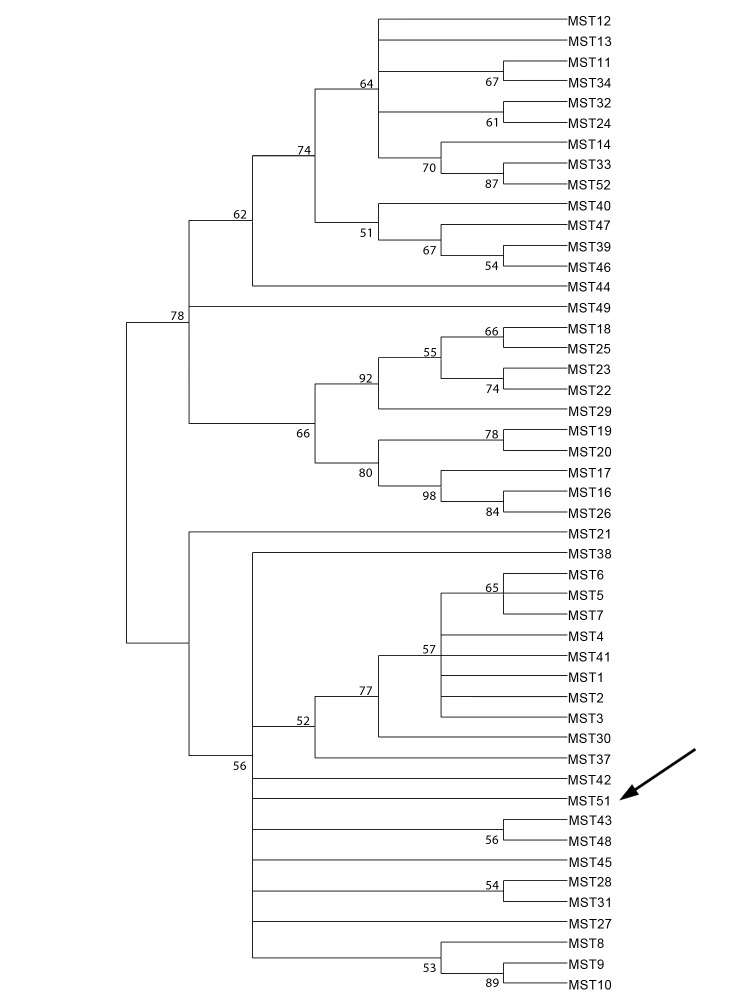
Neighbor-joining tree of *Coxiella burnetii* genotypes determined by multispacer sequence typing. Arrow indicates new genotype in Saudi Arabia.

The second case was detected in 2012 in a 13-year-old boy in Saudi Arabia who had tetralogy of Fallot, a prosthetic pulmonary valve, 2 intracardiac stents, and long-term fever. Serologic testing results were positive for *C. burnetii*; phase I titers for IgG, IgM, and IgA were 51,200, 400, and 200, respectively, and phase II titers were 102,400, 800, and 400, respectively. Whereas serum and blood samples were negative for *C. burnetii* by real-time PCR for the IS1111 and the IS30A spacers, the β-actin control was positive (cycle threshold <30). For this patient, we did not receive any material for culture. The patient was given 200 mg oral doxycycline daily and 200 mg oral hydroxychloroquine 3 times daily for 18 months.

To the best of our knowledge, before the 2 cases presented here, only 3 cases of Q fever endocarditis in Saudi Arabia have been described; all patients were from rural regions of Saudi Arabia and had an underlying valvulopathy (*4*–*6*). Moreover, Q fever was not immediately suspected, and as a result, 1 patient died (*6*). However, for 2 other patients, valve replacement was necessary (*4*,*5*). Q fever is prevalent in Saudi Arabia, and the very high prevalence of Q fever among camels was proposed as the reason Q fever is endemic among humans in Saudi Arabia (*2*,*3*). Camels were also suspected as the probable source of acute Q fever in US soldiers returning from Saudi Arabia (*10*). We identified a new *C. burnetii* genotype in the aortic valve of the first patient reported here. More epidemiologic studies are needed to determine whether this novel genotype circulating in Saudi Arabia is endemic to Saudi Arabia and whether it plays a major role in the origin of Q fever and in public health in this country.

Our studies of Q fever cases in southern France have shown that >16% of patients with acute Q fever have endocarditis and that ≈16%–37% of patients with Q fever endocarditis could have had previous symptomatic acute Q fever infection (*1*). Thus, many cases of endocarditis might be avoided if patients with acute Q fever receive antimicrobial drugs as prophylaxis (*1*). For patients >40 years of age, transthoracic echocardiography should be performed because of the increased prevalence of valvulopathy and Q fever endocarditis in this population (*9*). As a result, more studies are needed to determine whether our data can affect local clinical practice.

## References

[1] Million M, Walter G, Thuny F, Habib G, Raoult D. Evolution from acute Q fever to endocarditis is associated with underlying valvulopathy and age and can be prevented by prolonged antibiotic treatment. Clin Infect Dis. 2013;57:836–44. 10.1093/cid/cit419 23794723

[2] Gelpi AP. Q fever in Saudi Arabia. Am J Trop Med Hyg. 1966;15:784–98 . 5950521

[3] Greth A, Calvez D, Vassart M, Lefevre PC. Serological survey for bovine bacterial and viral pathogens in captive Arabian oryx (*Oryx leucoryx* Pallas, 1776). Rev Sci Tech. 1992;11:1163–8 . 1305861 10.20506/rst.11.4.652

[4] Ross PJ, Jacobson J, Muir JR. Q fever endocarditis of porcine xenograft valves. Am Heart J. 1983;105:151–3. 10.1016/0002-8703(83)90293-4 6849229

[5] al-Hajjar S, Hussain Qadri SM, al-Sabban E, Jager C. *Coxiella burnetii* endocarditis in a child. Pediatr Infect Dis J. 1997;16:911–3. 10.1097/00006454-199709000-00020 9306492

[6] Saginur R, Silver SS, Bonin R, Carlier M, Orizaga M. Q-fever endocarditis. CMAJ. 1985;133:1228–30 . 4063935 PMC1346577

[7] Angelakis E, Richet H, Rolain JM, La SB, Raoult D. Comparison of real-time quantitative PCR and culture for the diagnosis of emerging rickettsioses. PLoS Negl Trop Dis. 2012;6:e1540. 10.1371/journal.pntd.0001540 22413026 PMC3295807

[8] Angelakis E, Million M, D'Amato F, Rouli L, Richet H, Stein A, Q fever and pregnancy: disease, prevention, and strain specificity. Eur J Clin Microbiol Infect Dis. 2013;32:361–8 . 10.1007/s10096-012-1750-3 23052984

[9] Million M, Walter G, Bardin N, Camoin L, Giorgi R, Bongrand P, Immunoglobulin G anticardiolipin antibodies and progression to Q fever endocarditis. Clin Infect Dis. 2013;57:57–64 . 10.1093/cid/cit191 23532474

[10] Hussein MF, Al-Khalifa IM, Aljumaah RS, Gar Elnabi A, Mohammed OB, Omer SA, Serological prevalence of *Coxiella burnetii* in captive wild ruminants in Saudi Arabia. Comp Clin Pathol. 2012;21:33–8. 10.1007/s00580-010-1061-y

